# Translation and Assessment of Structural Validity and Internal Consistency of the Polish Health Literacy Questionnaire (HLQ) in Health Professions Students

**DOI:** 10.3390/medicina62091801

**Published:** 2026-09-18

**Authors:** Agnieszka Lipiak, Monika Karasiewicz, Barbara Gawłowska, Rafał Staszewski, Sylwia Wiśniewska-Leśków, Melanie Hawkins, Richard H. Osborne, Ewelina Chawłowska

**Affiliations:** 1 Department of Preventive Medicine, Poznan University of Medical Sciences, 60-781 Poznan, Poland; 2 Department of Foreign Language Teaching, Poznan University of Medical Sciences, 61-806 Poznan, Poland; 3 Global Health and Equity Development Hub, La Trobe Rural Health School, La Trobe University, Bendigo, VIC 3550, Australia

**Keywords:** Health Literacy Questionnaire (HLQ), health literacy measurement, psychometrics, validation, university students

## Abstract

*Background and Objectives*: Assessing health literacy requires valid measurement tools that capture its multidimensional nature. The nine-scale Health Literacy Questionnaire (HLQ) is globally used for this purpose. This study reports on the initial psychometric assessment of the Polish version of the HLQ. *Materials and Methods*: The HLQ was translated using the developers’ Translation Integrity Procedure and administered to 918 health professions students in a cross-sectional survey. Confirmatory factor analyses (CFA) and reliability tests were conducted. *Results*: One-factor CFAs generally supported the intended structure of the individual HLQ scales, although some variation in model fit was observed across scales. A full, restrictive nine-factor CFA supported the original nine-factor model, with good fit indices: χ^2^_WLSMV_ (866 df) = 1752.08, *p* < 0.0001, CFI = 0.973, TLI = 0.970, SRMR = 0.045, RMSEA = 0.035. Factor loadings were generally acceptable (≥0.50), with only two items below the threshold. Internal consistency of scales was good to acceptable in all but one domain marginally lower than desired: “Appraisal of health information” (Cronbach’s α = 0.69, composite reliability 0.68). *Conclusions*: The findings provide initial evidence supporting structural validity and internal consistency of the Polish HLQ in this sample. Further testing in other Polish populations is recommended.

## 1. Introduction

Since its first use in 1959 [1], the term “health literacy” (HL) has become one of the most important concepts in health promotion and public health. In recent years, over a thousand HL-related research papers have been published annually [2]. Numerous models and definitions of HL have been developed [3,4,5]. The World Health Organization (WHO) defines HL as “people’s knowledge, confidence and comfort—which accumulate through daily activities and social interactions and across generations—to access, understand, appraise, remember and use information about health and health care, for the health and well-being of themselves and those around them” [6]. This definition stresses the complex, multidimensional character of the concept.

HL is recognised as a critical determinant of health [7], vital to achieving not only personal health and well-being, but also health equity [8]. Research suggests that “low” or “inadequate” HL is associated with lower use of preventive care, more hospitalisations, and—in certain populations with poorer overall health status—higher mortality and lower quality of life [9,10,11]. In addition, it is more often identified in groups with low socioeconomic status, low education levels, older age and minority status [12,13]. The WHO’s Shanghai Declaration on promoting health prioritised HL development in all populations and educational settings [7]. To support this goal, reliable HL measurement is needed to assess population profiles and inform the design of effective and equitable health policies.

To this aim, multiple HL measurement tools have been developed for various populations [14]. For adults, a wide range of instruments is available, varying considerably in their scope and conceptual focus. Some instruments, for instance, the Newest Vital Sign [15], assess specific, often very narrow, HL dimensions [14,16], such as nutrition-related reading and numeracy skills. Other tools, such as the HLS-EU-Q [17], strive to measure a wider range of capabilities, including the respondent’s perceived skill at information seeking, communication, or comprehension [17]. Such questionnaires were used in studies on Polish population samples [18,19] and in cross-country comparisons [13], where HL was typically reported as a mean test score interpreted as adequate or inadequate HL in the population. While this approach was intended to identify at-risk groups, it may also contribute to stigmatising them. It also fails to capture the multidimensional character of HL, i.e., overlooks the fact that individuals may struggle in certain aspects of HL and excel in others. In addition, it may lead to developing one-size-fits-all health interventions that primarily suit the average person and fail to adequately support individuals with low or high levels of HL across different capabilities.

An alternative approach would be to measure multiple HL dimensions, focusing on strengths and potential solutions rather than deficits. Based on this approach, Osborne et al. [20] developed the 44-item Health Literacy Questionnaire (HLQ) in Australia. The questionnaire measures HL across nine distinct scales and uses separate scale scores to profile individuals and communities. As a result, the HLQ is suitable not only for identifying specific HL needs and strengths within populations, but also for guiding the development of targeted interventions. These qualities have driven its global adoption and established it as a key instrument in National Health Literacy Demonstration Projects supported by the WHO for the prevention and control of non-communicable diseases [21,22].

However, despite its widespread use internationally, a Polish translation of the HLQ has not been available. To address this gap, we undertook the translation and psychometric testing of the instrument. Two samples were selected for the assessment: health professions students and primary care patients. This paper presents the results of the initial psychometric analysis from the former group.

Health professions students were chosen for several reasons. Firstly, understanding HL is crucial for their future work, yet our teaching experience shows that even senior students often lack awareness of HL and its importance. Secondly, research indicates that student communities have unique HL needs [23]. Medical students’ HL profiles differ from both the general population and students of other disciplines [24,25] as well as across health majors [26,27]. This may necessitate adjusting university curricula for the sake of the students themselves and for the benefit of their future patients, whose HL needs they will be responding to [28]. Thirdly, to the best of our knowledge, evidence on HL among Polish health professions university students is scarce [29,30].

Thus, the aim of this study was to report on the Polish translation of the HLQ and to provide an initial psychometric assessment of its internal structure and reliability (internal consistency) in a sample of health professions students.

## 2. Materials and Methods

### 2.1. Participants and Setting

An online cross-sectional survey was carried out in the academic year 2023/24. Participants were university students in health professions courses conducted in Polish at the Poznan University of Medical Sciences (PUMS), one of the largest public medical universities in Poland. Convenience sampling was used. All adult students enrolled in full-time Bachelor’s and Master’s degree programmes were eligible to participate. Students were recruited through one-time announcements made during breaks between classes and invited to complete an online questionnaire. As a complementary recruitment strategy, posters containing a QR code linking to the survey were displayed in one of the main university buildings. Participation was voluntary, and students could decline or discontinue participation at any time by leaving the survey. All items except open-ended questions were set as mandatory; therefore, only fully completed questionnaires could be submitted and included in the analysis. Consequently, no item-level or question-level missing data were present among completed responses.

### 2.2. Ethics

Before accessing the questionnaire, participants were made aware of the aims of the study and of their right to withdraw from it at any time without any consequences. It was stressed that participation was voluntary and anonymous. The survey was conducted among adult students who provided electronic informed consent. No directly identifying personal information (e.g., names, student IDs, email addresses, IP addresses) was collected by the research team. Access to the survey data was restricted to the research team and password-protected. All procedures complied with the ethical principles of the Declaration of Helsinki. Prior to data collection, the ethical feasibility of the study was reviewed by the PUMS Bioethics Committee. According to the decision of the Committee No. KB—807/23 of 11 October 2023, the study did not constitute a medical experiment under Polish law and therefore did not require ethical approval.

### 2.3. The Tool

The survey questionnaire included the HLQ and additional sociodemographic questions. The HLQ was originally developed in 2013 in English [20]. The instrument consists of 44 items in nine domains (scales), each representing a different set of health literacy actions. The scales are:

1. Feeling understood and supported by healthcare providers.
2. Having sufficient information to manage my health.
3. Actively managing my health.
4. Social support for health.
5. Appraisal of health information.
6. Ability to actively engage with healthcare providers.
7. Navigating the healthcare system.
8. Ability to find good health information.
9. Understand health information well enough to know what to do.

In the first part of the HLQ comprising scales 1–5, respondents are asked to agree or disagree with statements, and responses are scored on a 4-point scale from “strongly disagree” to “strongly agree”. In the second part with scales 6–9, respondents determine the difficulty of certain tasks, and responses are scored on a 5-point scale from “cannot do or always difficult” to “always easy”. HLQ scale scores interpreted broadly as high and low are linked to scale score descriptions provided in the HLQ development paper [20], and are referred to as HL strengths and challenges [20,31]. The tool was demonstrated to have robust psychometric properties [32]. As of 2024, it was translated into more than 40 languages and psychometrically tested in multiple studies across diverse populations, showing consistent reliability across countries and languages [33].

### 2.4. The Translation of the HLQ

A licence to use and translate the HLQ had been obtained from the HLQ authors (licence no. TL1902IA), including the requirement to follow the Translation Integrity Procedure developed by the authors [34]. The procedure prioritises construct equivalence and linguistic simplicity.

As part of the procedure, AL translated the HLQ into Polish, with particular attention to item intent descriptions also provided by the authors. They specify both the construct element that each item is intended to measure and the level of health literacy trait reflected by different response options. The translation was subsequently reviewed by the second translator (SWL). Both were native speakers of Polish and professional translators, one with experience in public health research. Three detailed discussions followed between the two translators and a public health researcher fluent in English and well-versed in health education (EC) to explore any discrepancies. To resolve any remaining issues, translated item variants were piloted with six community members from different backgrounds (i.e., potential future respondents).

As a result, a provisional target translation was prepared. Each item was then blindly back-translated into English by a native speaker of English fluent in Polish, holding a Master’s degree in Slavic Philology and in Intercultural Studies. The provisional forward translation, the back translation, and all translators’ comments were reviewed by the HLQ author (RHO), who provided written feedback. Finally, the whole team met to discuss discrepancies in detail in a group cognitive interview with HLQ authors. All issues were resolved, and a final consensus HLQ version ready for pre-testing was prepared.

To pre-test the questionnaire, cognitive interviews were conducted following a standardised HLQ cognitive interviewing guide provided by the HLQ authors. Participants, primary care patients (n = 6) and university students (n = 6), were selected to achieve sociodemographic diversity in terms of age, education, and residence (urban vs. rural). Four members of the research team conducted face-to-face interviews (AL: 8, SWL: 3, MK: 1) after jointly reviewing the interview guide to ensure a consistent approach. To reduce participant burden, each respondent completed either Part 1 (23 items) or Part 2 (21 items) of the HLQ, resulting in each item being reviewed by six participants. Following independent questionnaire completion, retrospective probing was used to explore participants’ understanding of the items and the reasoning underlying their responses. Interview narratives were then compared with the HLQ item intent descriptions to evaluate conceptual equivalence between the Polish translation and the original instrument.

### 2.5. Statistical Analysis

PQStat v. 1.8.6 (PQStat Software, Poznan, Poland) was used for descriptive analyses.

Item difficulty, a term used here to express the propensity of individuals to endorse the higher ends of the HLQ scales, was estimated for items within their respective scales to assess the range of HL challenge that the items presented to respondents. More “difficult” items are those for which fewer respondents reported higher levels of agreement or ease of doing a task. For Part 1, item difficulty was determined as the proportion of “Strongly disagree” and “Disagree” responses as against “Agree” or “Strongly agree” responses. For Part 2, item difficulty was determined as the proportion of participants responding “Cannot do or always difficult”, “Usually difficult” and “Sometimes difficult” as against “Usually easy” and “Always easy” [35,36].

Subsequent analyses focused on evaluating the internal structure and consistency of the Polish HLQ, thereby replicating the procedures reported in the original HLQ development paper as an initial step in the validation process [20].

To measure reliability, Cronbach’s α and Raykov’s composite reliability were calculated for each HLQ domain.

The nine HLQ constructs were specified a priori, so confirmatory factor analysis (CFA) was conducted using R Studio version 2024.04.2.764 with the lavaan and semTools packages (version 0.6.18) [37,38]. First, a one-factor model was fitted to the data for each scale, followed by a nine-factor model using a restrictive estimation approach, which excluded cross-loadings or correlated residuals. A cut-off value of 0.5 was used to define factor loadings acceptable in CFA [39]. The response options were coded as ordinal variables (1–4 for the disagree/agree responses and 1–5 for the difficult/easy responses). The models were fitted with the use of the weighted least squares mean and variance adjusted (WLSMV) estimator and robust/scaled values. The CFA was based on polychoric correlation matrices, which are appropriate for ordinal variables. To assess the goodness of fit of the model, a number of fit statistics (χ^2^, comparative fit index—CFI, Tucker–Lewis index—TLI, standardised root mean residual—SRMR, and root mean square error of approximation—RMSEA) were calculated. The threshold values indicative of a “good fit” were CFI > 0.95, TLI > 0.95, RMSEA < 0.06, and SRMR ≤ 0.08 [40].

## 3. Results

### 3.1. Translation

The narratives collected during cognitive interviews demonstrated that all participants understood items as intended: responses aligned with item intent descriptions. Ten of the twelve interviewees perceived the questionnaire as repetitive. One person noticed, however, that two seemingly similar items prompted different responses. Four students found certain items abstract due to limited health system experience. Notably, two interviewees reported that completing the questionnaire made them reflect on their use of health services and health information. This suggests that, while HL may be unfamiliar to some Polish respondents, the wording of the HLQ is clear and understandable. As the interviewees engaged well with the items, no changes were made before psychometric testing.

### 3.2. Sample Characteristics

A total of 924 students initiated the survey. Of these, six declined participation by responding “no” to the consent question, and 918 agreed to participate and completed the questionnaire. The resulting sample size (n = 918) constituted about 16% of all full-time students at that time [41]. Among the 918 survey participants, the average age was 21.2 years (SD = 3.1). A majority of the respondents (80%) were female, consistent with national trends: women represented 76.4% of health professions students in Poland in 2023 [42]. A majority (64.3%) were first- or second-year students, with Medicine students constituting the largest group (39.1%). Nearly half (49.7%) reported good overall health and good economic status (46.5%). More detailed participant characteristics can be found in Table 1.

### 3.3. Item Difficulty

Difficulty of the items and scales of the HLQ is presented in Table 2. Scale-level difficulty was calculated as the arithmetic mean of the item-level difficulty values for all items within each scale.

In Part 1 of the questionnaire, Scale 4, social support for health, had the lowest difficulty (16.6%), and Scale 2, having sufficient information to manage my health, had the highest difficulty (37.3%). In the second part, Scale 9, understand health information well enough to know what to do, presented the lowest difficulty to the respondents (31.8%), while Scale 7, navigating the healthcare system, turned out to have the highest difficulty (53.7%).

Item difficulty showed considerable variation within most scales. In Part 1, the smallest, but still with a wide range of difficulty, was observed in Scale 1 (22.2–31.3%), and the largest was observed in Scale 5 (7.3–52.6%). In Part 2, the smallest variation was found in Scale 9 (23.8–41.8%), while the largest was in Scale 6 (13.0–66.5%).

The above data present difficulty levels using dichotomised values. A full account of percentages for all response categories, as well as floor and ceiling values, is provided in Tables S1 and S2 (Supplementary Material).

### 3.4. Internal Consistency of the Nine HLQ Factors

As presented in Table 3, internal consistency of HLQ scales measured with Cronbach’s α ranged from 0.69 (Scale 5) to 0.85 (Scale 1), demonstrating acceptable reliability for most scales, with Scale 5 slightly below the conventional 0.70 threshold. Composite reliability (CR), which better accounts for unequal factor loadings [43], produced similar estimates ranging from 0.68 for Scale 5 to 0.85 for Scale 1.

### 3.5. Confirmatory Factor Analysis

For comparability with the original [20] and translated HLQ studies [36,44,45,46,47,48,49,50], we used similar statistical methods in CFA.

For the one-factor models, seven scales demonstrated good fit (RMSEA < 0.06), including Scale 7, which approached the recommended cutoff (0.056). For Scales 2 and 3, some robust/scaled fit indices (including RMSEA, CFI, and TLI) were not available in the software output. This was likely related to the very low degrees of freedom of these models, which can affect the estimation and interpretation of robust fit indices. While this requires more cautious interpretation, low SRMR values and acceptable factor loadings in these scales provide support for their unidimensional structure.

For the nine-factor model, the fit was good: χ^2^_WLSMV_ (866 df) = 1752.08, *p* < 0.0001, CFI = 0.973, TLI = 0.970, SRMR = 0.045 and RMSEA = 0.035. Two items in total (one item in Scale 5 and one in Scale 9) had factor loadings below 0.50. Factor loadings for the rest of the items were acceptable, ranging from 0.53 to 0.85 (see Table 4).

Table 5 presents factor correlations spanning from 0.27 to 0.84. High correlations were observed only in Part 2 of the HLQ (≥0.64), with the highest being 0.84 between Scale 6, ability to actively engage with healthcare providers, and Scale 7, navigating the healthcare system, as well as 0.83 between Scale 8, ability to find good health information, and Scale 9, understand health information well enough to know what to do. To further explore the correlations, an additional assessment of discriminant validity was conducted using the heterotrait–monotrait ratio of correlations (HTMT). The HTMT values were 0.849 for scales 6 and 7 and 0.815 for scales 8 and 9. The values for all HLQ scales are presented in Table S3 (Supplementary Material).

## 4. Discussion

This study reports on the translation of the HLQ into Polish and presents the results of the initial psychometric testing of the translated version among health professions students. Qualitative data from cognitive interviews as well as quantitative data from psychometric tests indicate good fidelity of the Polish version to the original English HLQ. They also provide initial evidence of structural validity and internal consistency of the Polish HLQ and support its further evaluation for HL research and development in Poland.

The translation process followed the procedure provided by the authors of the parent instrument [34] and has been successfully used in numerous other translations [36,44,45,48,50]. The participants of cognitive interviews understood and responded to the items of the Polish HLQ in line with the item intents.

The first of the psychometric tests examined item difficulty within each scale, as the HLQ was designed to capture diverse HL challenges across nine domains [20]. Consistent with this design, the Polish HLQ demonstrated substantial variability in item difficulty, especially in Scale 6, ability to actively engage with healthcare providers (13.0–66.4%). Average scale difficulty also varied widely. Scale 4, social support for health, was relatively easy for students to endorse, possibly reflecting reliance on personal networks for health management among these generally healthy young adults. On the other hand, the high difficulty of Scale 7, navigating the healthcare system, could result from students’ limited experience with the system. This finding may indicate a need for greater emphasis on healthcare system navigation in health professions education and illustrates how the HLQ may help explore potential educational needs. While more in-depth research is necessary to explore these interpretations, the observed variability in item and scale difficulty suggests that the items capture different aspects of health literacy and vary in the level of challenge they present to respondents, as originally intended. This profile-based approach is a key advantage over instruments yielding a single score [16]. Individual HLQ scales are increasingly used in population studies to precisely determine HL needs in specific domains and to inform public health actions [51,52].

Next, internal consistency of HLQ scales was assessed. Cronbach’s α and composite reliability were acceptable or good (≥0.70), except for Scale 5, appraisal of health information, which had slightly lower values. The scale presented the lowest internal consistency in several other translations: Slovak [47], Dutch [48], Danish [36], and German [50]. The consistency of this finding across different language versions suggests that it may not be solely attributable to translation-related issues. Instead, it may reflect the inherent complexity of the construct assessed by this scale. Information appraisal requires individuals to evaluate the credibility, quality, and applicability of information obtained from multiple sources and may be more difficult for respondents to endorse consistently. Nevertheless, because some items address relatively abstract concepts, challenges related to semantic and conceptual equivalence across languages cannot be entirely excluded. Future research should examine the functioning of Scale 5 in diverse populations and cultural contexts to better understand the sources of its comparatively weaker psychometric performance. From a practical perspective, the finding suggests that although Scale 5 captures a relevant aspect of HL, its scores may be less precise and should be interpreted with caution.

A strict CFA without cross-loadings or correlated residuals demonstrated at least satisfactory fit across HLQ scales [40]. For the one-factor models, most scales showed good fit (RMSEA < 0.06). The higher values observed for Scales 2 and 3 may indicate sensitivity to contextual factors, given known limitations of RMSEA in models with small degrees of freedom [53], such as a small number of items in each scale and the use of one-factor models. The nine-factor model demonstrated good fit. Taken together, the evidence supports the nine-factor structure of the Polish HLQ consistent with the structure of the original questionnaire. At the item level, factor loadings ranged from 0.41 to 0.85. Only two items—one in Scale 5 and one in Scale 9—fell below the conventional 0.50 threshold acceptable in CFA of multifaceted constructs [39], representing a small proportion (4.5%) of the total item pool. Scale 5, appraisal of health information, with one item loading below 0.5 and Cronbach’s α = 0.69, may need further research in a more diverse sample to check item functioning.

Some scales of the HLQ showed high inter-factor correlations in Part 2 of the questionnaire: 0.84 between scales 6 and 7, and 0.83 between scales 8 and 9, indicating a potential lack of discriminant validity. However, the respective HTMT values were 0.849 and 0.815, that is slightly below the commonly applied threshold of 0.85 [54]. These findings indicate a relatively high degree of association between the corresponding domains, while also providing cautious evidence in support of their discriminant validity. Similar patterns in Part 2 were reported in the original HLQ development study [20] and in several studies testing HLQ translations [36,48,50,55]. Previous research suggests that these relationships may reflect a degree of causality between Part 2 domains or a presence of broader underlying dimensions [32,56]. It was hypothesised that all HLQ items may reflect a higher-order factor related to a sense of agency and efficacy in engaging with health information, with scales 6 (actively engaging with healthcare providers), 7 (navigating the healthcare system), and 8 (finding good health information) loading particularly strongly on this general dimension. At the same time, this general dimension does not fully account for variability across all domains, each probably reflecting its own independent factor [56].

Although the results discussed above are broadly consistent with previous HLQ research, several limitations should be taken into account. The study was based on a convenience sample drawn from a single medical university, and the recruitment strategy did not provide all eligible students with a comparable opportunity to participate. In addition, women and early-year students were overrepresented, and the overall participation rate was relatively low. These factors increase the risk of selection bias and limit generalisability of results across the student population. What is more, the study group consisted of young, generally healthy, Polish-speaking future health professionals, predominantly with favourable socioeconomic status. As a result, the sample may have represented individuals with fewer HL challenges than the wider population, leading to a more restricted range of item responses. This may have affected some psychometric estimates, including reliability estimates. Consequently, the psychometric properties and response patterns observed in this study may differ in populations with lower educational attainment, different socioeconomic characteristics, or a wider range of health literacy levels. Finally, while the results support the hypothesised nine-domain structure, high correlations between Part 2 scales indicate overlap and should be considered when interpreting separate scale scores. Future studies need to examine alternative factor structures, including higher-order models, and report formal indices of discriminant validity where appropriate. Therefore, further research using the HLQ in more diverse Polish samples, including populations more representative of the general population, is warranted.

The study also has notable strengths. The translation and subsequent validity testing of the HLQ followed well-established protocols, including the Translation Integrity Procedure with its emphasis on item intents, pilot testing and cognitive interviews, and restrictive confirmatory factor analyses. As a result, the findings contribute validity and reliability evidence about the Polish HLQ in this student population.

## 5. Conclusions

We examined the internal structure and internal consistency of the HLQ in health professions students at one university in Poland. Further psychometric testing is needed, including examination of discriminant validity among the HLQ’s nine factors, known groups validity, test–retest reliability, measurement error, and measurement invariance across relevant subgroups. Nevertheless, the study findings provide initial evidence of the structural validity and internal consistency of the Polish HLQ in this population and support its further evaluation in broader and more diverse populations. If confirmed in future studies, the Polish HLQ may become a useful instrument for health literacy research and practice in Poland.

## Figures and Tables

**Table 1:** Characteristics of the study sample (n = 918).

Variable		n (%)
Sex		
	Female	734 (79.96%)
	Male	184 (20.04%)
Age (years)		
	M (SD) = 21.22 (3.09)	
	18	45 (4.9%)
	19	184 (20.04%)
	20	240 (26.14%)
	21	111 (12.09%)
	22	106 (11.55%)
	23	145 (15.8%)
	24	30 (3.27%)
	25	22 (2.4%)
	26–54	35 (3.82%)
Programme of study		
	Medicine	359 (39.11%)
	Nursing	131 (14.27%)
	Midwifery	108 (11.76%)
	Dentistry	66 (7.19%)
	Public Health	60 (6.54%)
	Radiography	48 (5,23%)
	Medical Analytics	41(4,47%)
	Pharmaceutical Engineering	32 (3.49%)
	Dietetics	31 (3.38%)
	Other	42 (4.57%)
Year of study		
	1	250 (27.23%)
	2	340 (37.04%)
	3	92 (10.02%)
	4	187 (20.37%)
	5	45 (4.9%)
	6	4 (0.44%)
Self-reported health status		
	Good	456 (49.67%)
	Fairly good	328 (35.73%)
	Average	112 (12.2%)
	Unsatisfactory	18 (1.96%)
	Poor	4 (0.44%)
Have you been diagnosed with a chronic condition?		
	Yes	232 (25.27%)
	No	686 (74.73%)
Number of diagnosed chronic conditions (based on an open-ended question)		
	0	694 (75.6%)
	1	141 (15.36%)
	2	58 (6.32%)
	3 or more	25 (2.72%)
Self-reported economic status		
	Excellent	202 (22.00%)
	Good	427 (46.51%)
	Average	267 (29.08%)
	Poor	17 (1.85%)
	Very poor	5 (0.54%)

M—mean; SD—standard deviation.

**Table 2:** Mean scores and difficulty of Polish Health Literacy Questionnaire items and scales in the study sample (n = 918).

Part/Scale/Item	M (SD)	Difficulty (%)
Part 1 (scores from 1 to 4 points)		
1. Feeling understood and supported by healthcare providers	2.96 (0.64)	24.78
I have at least one healthcare provider who…	2.93 (0.89)	31.26
I have at least one healthcare provider I can…	2.99 (0.76)	22.77
I have the healthcare providers I need…	2.97 (0.73)	22.22
I can rely on at least one…	2.95 (0.70)	22.88
2. Having sufficient information to manage my health	2.71 (0.54)	37.34
I feel I have good information about health…	3.11 (0.50)	6.64
I have enough information to help me deal…	2.65 (0.75)	41.29
I am sure I have all the information I…	2.52 (0.72)	50.98
I have all the information I need to…	2.54 (0.75)	50.44
3. Actively managing my health	2.81 (0.54)	30.96
I spend quite a lot of time actively managing…	2.66 (0.73)	42.48
I make plans for what I need to do to be…	2.84 (0.71)	30.17
Despite other things in my life, I make time…	2.70 (0.72)	35.40
I set my own goals about health and fitness	2.90 (0.65)	22.98
There are things that I do regularly…	2.93 (0.68)	23.75
4. Social support for health	3.12 (0.48)	16.62
I can get access to several people who…	3.43 (0.64)	5.23
When I feel ill, the people around me really…	2.60 (0.70)	42.37
If I need help, I have plenty of people I…	3.02 (0.68)	18.74
I have at least one person…	3.29 (0.63)	6.43
I have strong support from…	3.25 (0.67)	10.35
5. Appraisal of health information	2.93 (0.43)	23.27
I compare health information from different…	3.23 (0.60)	7.30
When I see new information about health, I…	2.95 (0.63)	21.46
I always compare health information from…	2.96 (0.61)	19.28
I know how to find out if the health…	3.03 (0.61)	15.69
I ask healthcare providers about the quality…	2.47 (0.78)	52.61
Part 2 (scores from 1 to 5 points)		
6. Ability to actively engage with healthcare providers	3.43 (0.63)	43.40
Make sure that healthcare providers understand…	3.13 (0.82)	66.45
Feel able to discuss your health concerns with a…	3.48 (0.85)	12.96
Have good discussions about your health…	3.47 (0.88)	49.46
Discuss things with healthcare providers…	3.44 (0.85)	51.09
Ask healthcare providers questions to get…	3.65 (0.84)	37.04
7. Navigating the healthcare system	3.35 (0.61)	53.72
Find the right healthcare	3.07 (0.87)	68.74
Get to see the healthcare providers you need to	3.18 (0.92)	62.75
Decide which healthcare provider you need…	3.66 (0.85)	36.06
Make sure you find the right place to get…	3.50 (0.85)	46.62
Find out what healthcare services you are…	3.18 (0.92)	61.76
Work out what the best care is for you	3.51 (0.76)	46.41
8. Ability to find good health information	3.74 (0.55)	33.07
Find information about health problems	3.67 (0.74)	35.29
Find health information from several…	3.84 (0.73)	26.58
Get information about health so you are…	3.55 (0.76)	44.66
Get health information in words you…	3.90 (0.75)	26.47
Get health information by yourself	3.75 (0.74)	32.35
9. Understand health information well enough to know what to do	3.77 (0.55)	31.79
Confidently fill medical forms in the correct…	3.78 (0.83)	31.92
Accurately follow the instructions from…	3.63 (0.77)	41.83
Read and understand written health…	3.84 (0.79)	28.54
Read and understand all the information on…	3.74 (0.95)	32.90
Understand what healthcare providers are…	3.88 (0.70)	23.75

M—mean; SD—standard deviation. Items are truncated; full items are available from the authors of the original HLQ.

**Table 3:** Cronbach’s α and composite reliability values of the nine Polish Health Literacy Questionnaire scales together with 95% confidence intervals.

Scale	1	2	3	4	5	6	7	8	9
Cronbach’s α (95% CI)	0.85 (0.83–0.87)	0.80 (0.77–0.82)	0.84 (0.82–0.85)	0.77 (0.74–0.80)	0.69 (0.65–0.73)	0.80 (0.77–0.82)	0.79 (0.77–0.82)	0.79 (0.77–0.82)	0.70 (0.67–0.73)
CR (95% CI)	0.85 (0.83–0.87)	0.79 (0.75–0.82)	0.83 (0.81–0.85)	0.77 (0.73–0.79)	0.68 (0.63–0.72)	0.80 (0.77–0.82)	0.80 (0.77–0.82)	0.78 (0.75–0.81)	0.71 (0.67–0.74)

CR—composite reliability; CI—confidence interval.

**Table 4:** Factor loadings of particular items and model fit indices of the nine scales in the Polish Health Literacy Questionnaire.

Scale/Item	Factor Loadings (Part 1)					Factor Loadings (Part 2)			
	Scale 1	Scale 2	Scale 3	Scale 4	Scale 5	Scale 6	Scale 7	Scale 8	Scale 9
1. Feeling understood and supported by healthcare providers χ^2^_WLSMV_ (2) = 5.791, *p* = 0.055, CFI = 1.000, TLI = 1.002, SRMR = 0.0104, RMSEA = 0.019 (90% CI = 0.000–0.037)									
1.2	0.644								
1.8	0.795								
1.17	0.816								
1.22	0.845								
2. Having sufficient information to manage my health χ^2^_WLSMV_ (2) = N/A, *p* = N/A, CFI = N/A, TLI = N/A, SRMR = 0.008, RMSEA = N/A									
1.1		0.664							
1.10		0.668							
1.14		0.703							
1.23		0.786							
3. Actively managing my health χ^2^_WLSMV_ (5) = N/A, *p* = N/A, CFI = N/A, TLI = N/A, SRMR = 0.016, RMSEA = N/A									
1.6			0.806						
1.9			0.629						
1.13			0.715						
1.18			0.642						
1.21			0.729						
4. Social support for health χ^2^_WLSMV_ (5) = 17.981, *p* = 0.003, CFI = 0.996, TLI = 0.992, SRMR = 0.024, RMSEA = 0.029 (90% CI = 0.015–0.044)									
1.3				0.642					
1.5				0.568					
1.11				0.725					
1.15				0.600					
1.19				0.626					
5. Appraisal of health information χ^2^_WLSMV_ (5) = 8.120, *p* = 0.150, CFI = 1.000, TLI = 1.004, SRMR = 0.018, RMSEA = 0.017 (90% CI = 0.000–0.037)									
1.4					0.540				
1.7					0.534				
1.12					0.558				
1.16					0.755				
1.20					0.414				
6. Ability to actively engage with healthcare providers χ^2^_WLSMV_ (5) = 11.644, *p* = 0.040, CFI = 1.000, TLI = 1.003, SRMR = 0.019, RMSEA = 0.020 (90% CI = 0.004–0.035)									
2.2						0.586			
2.4						0.622			
2.7						0.716			
2.15						0.700			
2.20						0.699			
7. Navigating the healthcare system χ^2^_WLSMV_ (9) = 78.936, *p* < 0.001, CFI = 0.988, TLI = 0.980, SRMR = 0.045, RMSEA = 0.056 (90% CI = 0.045–0.068)									
2.1							0.643		
2.8							0.628		
2.11							0.621		
2.13							0.673		
2.16							0.517		
2.19							0.693		
8. Ability to find good health information χ^2^_WLSMV_ (5) = 22.164, *p* < 0.001, CFI = 0.997, TLI = 0.995, SRMR = 0.029, RMSEA = 0.037 (90% CI = 0.022–0.054)									
2.3								0.617	
2.6								0.570	
2.10								0.714	
2.14								0.646	
2.18								0.724	
9. Understand health information well enough to know what to do χ^2^_WLSMV_ (5) = 20.560, *p* = 0.001, CFI = 0.994, TLI = 0.988, SRMR = 0.030, RMSEA = 0.040 (90% CI = 0.023–0.058)									
2.5									0.547
2.9									0.459
2.12									0.625
2.17									0.588
2.21									0.649

χ^2^—chi-square test statistic; WLSMV—weighted least squares mean and variance adjusted estimator; *p*—*p*-value; CFI—comparative fit index; CI—confidence interval; TLI—Tucker–Lewis index; SRMR—standardised root mean residual; RMSEA—root mean square error of approximation; N/A—robust/scaled fit index could not be estimated for this model.

**Table 5:** Factor correlations of the nine factors of the Polish Health Literacy Questionnaire.

		Scale 1	Scale 2	Scale 3	Scale 4	Scale 5	Scale 6	Scale 7	Scale 8
HLQ Part 1 (Scales 1–5)	Scale 2	0.327							
	Scale 3	0.277	0.448						
	Scale 4	0.524	0.344	0.377					
	Scale 5	0.412	0.553	0.523	0.401				
HLQ Part 2 (Scales 6–9)	Scale 6	0.506	0.362	0.274	0.403	0.325			
	Scale 7	0.440	0.401	0.308	0.419	0.345	0.836		
	Scale 8	0.292	0.488	0.306	0.300	0.551	0.636	0.715	
	Scale 9	0.282	0.438	0.349	0.305	0.507	0.679	0.726	0.831

Each factor represents a scale: 1. Feeling understood and supported by healthcare providers; 2. Having sufficient information to manage my health; 3. Actively managing my health; 4. Social support for health; 5. Appraisal of health information; 6. Ability to actively engage with healthcare providers; 7. Navigating the healthcare system; 8. Ability to find good health information; 9. Understanding health information well enough to know what to do.

## Data Availability

The data presented in this study are available from the corresponding author upon reasonable request. The data are not publicly available due to privacy and ethical restrictions.

## Supplementary Materials

The following supporting information can be downloaded at https://www.mdpi.com/article/10.3390/medicina62091801/s1. Table S1. Percentages for all response categories of Health Literacy Questionnaire items. Table S2. Floor and ceiling effects in Health Literacy Questionnaire scales. Table S3. Assessment of discriminant validity using the heterotrait-monotrait ratio among the nine Health Literacy Questionnaire scales.

## Abbreviations

The following abbreviations are used in this manuscript:

CFA	Confirmatory factor analysis
CFI	Comparative fit index
CI	Confidence interval
CR	Composite reliability
HL	Health literacy
HLQ	Health Literacy Questionnaire
HTMT	Heterotrait–monotrait ratio of correlations
M	Mean
PUMS	Poznan University of Medical Sciences
RMSEA	Root mean square error of approximation
SD	Standard deviation
SRMR	Standardised root mean residual
TLI	Tucker–Lewis index
WHO	World Health Organization
WLSMV	Weighted least squares mean and variance adjusted estimator
χ^2^	chi-square test statistic
