# Supplementary material for: Translation and Assessment of Structural Validity and Internal Consistency of the Polish Health Literacy Questionnaire (HLQ) in Health Professions Students
Source: Medicina (Kaunas). 2026 Sep 18;62(9):1801. doi: 10.3390/medicina62091801 (PMC13608996; doi:10.3390/medicina62091801)
Supplement: Supplementary file 1 [file medicina-62-01801-s001.zip › medicina-4545483-supplementary.pdf]

Supplementary Material

Table S1. Percentages for all response categories of Health Literacy Questionnaire items.

**Part 1**	**Strongly agree (ceiling effect)**	**Agree**	**Disagree**	**Strongly disagree (floor effect)**	
HLQ1.1	167 (18.19%)	690 (75.16%)	56 (6.1%)	5 (0.54%)	
HLQ1.2	279 (30.39%)	352 (38.34%)	232 (25.27%)	55 (5.99%)	
HLQ1.3	453 (49.35%)	417 (45.42%)	34 (3.7%)	14 (1.53%)	
HLQ1.4	289 (31.48%)	562 (61.22%)	60 (6.54%)	7 (0.76%)	
HLQ1.5	68 (7.41%)	461 (50.22%)	341 (37.15%)	48 (5.23%)	
HLQ1.6	110 (11.98%)	418 (45.53%)	358 (39%)	32 (3.49%)	
HLQ1.7	154 (16.78%)	567 (61.76%)	191 (20.81%)	6 (0.65%)	
HLQ1.8	231 (25.16%)	478 (52.07%)	178 (19.39%)	31 (3.38%)	
HLQ1.9	149 (16.23%)	492 (53.59%)	258 (28.1%)	19 (2.07%)	
HLQ1.10	103 (11.22%)	436 (47.49%)	330 (35.95%)	49 (5.34%)	
HLQ1.11	205 (22.33%)	541 (58.93%)	156 (16.99%)	16 (1.74%)	
HLQ1.12	147 (16.01%)	594 (64.71%)	170 (18.52%)	7 (0.76%)	
HLQ1.13	94 (10.24%)	498 (54.25%)	281 (30.61%)	44 (4.79%)	
HLQ1.14	78 (8.5%)	371 (40.41%)	420 (45.75%)	48 (5.23%)	
HLQ1.15	340 (37.04%)	519 (56.54%)	44 (4.79%)	15 (1.63%)	
HLQ1.16	177 (19.28%)	597 (65.03%)	136 (14.81%)	8 (0.87%)	
HLQ1.17	205 (22.33%)	509 (55.45%)	177 (19.28%)	27 (2.94%)	
HLQ1.18	138 (15.03%)	569 (61.98%)	194 (21.13%)	17 (1.85%)	
HLQ1.19	340 (37.04%)	483 (52.61%)	83 (9.04%)	12 (1.31%)	
HLQ1.20	78 (8.5%)	357 (38.89%)	398 (43.36%)	85 (9.26%)	
HLQ1.21	166 (18.08%)	534 (58.17%)	203 (22.11%)	15 (1.63%)	
HLQ1.22	185 (20.15%)	523 (56.97%)	189 (20.59%)	21 (2.29%)	
HLQ1.23	93 (10.13%)	362 (39.43%)	415 (45.21%)	48 (5.23%)	
**Part 2**	**Always easy (ceiling effect)**	**Usually easy**	**Sometimes difficult**	**Usually difficult**	**Cannot do (floor effect)**
HLQ2.1	40 (4.36%)	247 (26.91%)	390 (42.48%)	223 (24.29%)	18 (1.96%)
HLQ2.2	23 (2.51%)	285 (31.05%)	416 (45.32%)	176 (19.17%)	18 (1.96%)
HLQ2.3	82 (8.93%)	512 (55.77%)	265 (28.87%)	56 (6.1%)	3 (0.33%)
HLQ2.4	78 (8.5%)	411 (44.77%)	310 (33.77%)	108 (11.76%)	11 (1.2%)
HLQ2.5	161 (17.54%)	464 (50.54%)	226 (24.62%)	61 (6.64%)	6 (0.65%)
HLQ2.6	137 (14.92%)	537 (58.5%)	207 (22.55%)	34 (3.7%)	3 (0.33%)
HLQ2.7	95 (10.35%)	369 (40.2%)	339 (36.93%)	101 (11%)	14 (1.53%)
HLQ2.8	60 (6.54%)	282 (30.72%)	361 (39.32%)	193 (21.02%)	22 (2.4%)
HLQ2.9	100 (10.89%)	434 (47.28%)	325 (35.4%)	58 (6.32%)	1 (0.11%)
HLQ2.10	75 (8.17%)	433 (47.17%)	341 (37.15%)	64 (6.97%)	5 (0.54%)
HLQ2.11	119 (12.96%)	468 (50.98%)	240 (26.14%)	81 (8.82%)	10 (1.09%)
HLQ2.12	165 (17.97%)	491 (53.49%)	215 (23.42%)	42 (4.58%)	5 (0.54%)
HLQ2.13	86 (9.37%)	404 (44.01%)	322 (35.08%)	92 (10.02%)	14 (1.53%)
HLQ2.14	180 (19.61%)	494 (53.81%)	212 (23.09%)	29 (3.16%)	2 (0.22%)
HLQ2.15	78 (8.5%)	371 (40.41%)	358 (39%)	96 (10.46%)	15 (1.63%)
HLQ2.16	54 (5.88%)	297 (32.35%)	352 (38.34%)	189 (20.59%)	26 (2.83%)
HLQ2.17	184 (20.04%)	432 (47.06%)	193 (21.02%)	94 (10.24%)	15 (1.63%)
HLQ2.18	117 (12.99%)	487 (54.05%)	260 (28.86%)	33 (3.66%)	4 (0.44%)
HLQ2.19	59 (6.43%)	433 (47.17%)	351 (38.24%)	67 (7.3%)	8 (0.87%)
HLQ2.20	114 (12.42%)	464 (50.54%)	263 (28.65%)	61 (6.64%)	16 (1.74%)
HLQ2.21	137 (14.92%)	563 (61.33%)	194 (21.13%)	18 (1.96%)	6 (0.65%)

Table S2. Floor and ceiling effects in Health Literacy Questionnaire scales.

	**Floor effect**	**Ceiling effect**
Scale 1	0.76%	10.89%
Scale 2	0.33%	3.27%
Scale 3	0.22%	3.70%
Scale 4	0.44%	4.03%
Scale 5	0.22%	2.29%
Scale 6	0.00%	1.20%
Scale 7	0.00%	0.76%
Scale 8	0.00%	2.83%
Scale 9	0.00%	2.07%

Table S3. Assessment of discriminant validity using the heterotrait-monotrait ratio among the nine Health Literacy Questionnaire scales.

	Scale 1	Scale 2	Scale 3	Scale 4	Scale 5	Scale 6	Scale 7	Scale 8	Scale 9
Scale 1	1.000								
Scale 2	0.343	1.000							
Scale 3	0.267	0.460	1.000						
Scale 4	0.517	0.353	0.369	1.000					
Scale 5	0.429	0.532	0.547	0.388	1.000				
Scale 6	0.506	0.357	0.212	0.406	0.325	1.000			
Scale 7	0.439	0.404	0.303	0.417	0.330	0.849	1.000		
Scale 8	0.286	0.492	0.295	0.299	0.524	0.624	0.710	1.000	
Scale 9	0.279	0.448	0.349	0.306	0.452	0.685	0.746	0.815	1.000
